# “Stop, Think, and Appreciate”: A Qualitative Exploration of a Challenge Coin Suicide Prevention Intervention among Farmers

**DOI:** 10.1007/s10597-025-01509-1

**Published:** 2025-08-20

**Authors:** Jeanne M. Ward, Melissa Perkins, John R. Blosnich

**Affiliations:** 1 Suzanne Dworak-Peck School of Social Work, University of Southern California, 669 W 34th St, Los Angeles, CA 90089 USA; 2 School of Nursing, Indiana University, 600 Barnhill Drive, Indianapolis, IN 46202 USA; 3 Center for Health Equity Research and Promotion, VA Pittsburgh Healthcare System, Pittsburgh, PA 15240 USA

**Keywords:** Agriculture, Farming, Suicide, Suicide prevention, Mental health, Qualitative evaluation

## Abstract

Farmers are at increased risk for suicide compared to the general population, with estimates 56% higher among males in agriculture/forestry/fishing roles than the male working population. This study explored a farmer-developed suicide prevention intervention using an adapted military challenge coin for agriculture. An agricultural community member shared a message of appreciation with the farmer recipient. Farmers recently receiving a challenge coin were purposively sampled. Semi-structured interviews via telephone/videoconference explored farmers’ challenge coin experiences and perceptions. Interviews were transcribed verbatim and de-identified before thematic coding. Participants (*n* = 14) were aged 28–68 years. All interviewees were non-Hispanic White, and 71% had off-farm jobs. Themes included the reception of the challenge coin, feelings elicited, and protective nature of the challenge coin against suicide, encouraging farmer connectedness and demonstrating appreciation. These data provide initial exploration of challenge coins adapted for farmer suicide prevention, developed within a farming community. Additional research regarding the impact is needed.

**Supplementary Information:**

The online version contains supplementary material available at 10.1007/s10597-025-01509-1.

## Introduction

Suicide is the eleventh leading cause of death in the United States, and it increased 33% between 2001 and 2018 (Garnett & Curtin, 2023). The suicide rate among farmers and individuals working in agriculture is higher than that of the general population (Miller & Rudolphi, 2022; Norrod et al., 2023; Peterson et al., 2020), necessitating new suicide prevention tactics. While the overall working male suicide rate was 32.0 per 100,000, among males working in agriculture, fishing, and forestry, it was 56% higher, with a suicide rate of 49.9 per 100,000 in 2021 (Sussell et al., 2023). Further, non-metropolitan suicide rates climbed from 16.4 per 100,000 in 2002 to 20.1 per 100,000 in 2022, a rate of increase higher than metropolitan suicides (Centers for Disease Control and Prevention [CDC], 2023). Farmer/rancher suicide decedents in the U.S. are more likely than the general population to be males over age 60 and White (Miller & Rudolphi, 2022).

Farmers face stressors related to finances, unpredictable weather, and geographic isolation (Hagen et al., 2019; Purc-Stephenson et al., 2023), which can contribute to poor mental health and ultimately higher occupational suicide rates (Joo & Roh, 2016; Purc-Stephenson et al., 2023; Reed & Claunch, 2020). Belongingness, social support, and occupational appreciation are vital factors that help protect against farmer distress and suicidality, as they help farmers combat isolation and potentially low morale that may be attributable to the uncontrollable aspects of farming (e.g., weather), dynamic commodity pricing, and physical labor demands (Bjornestad et al., 2021; Liang, 2021; McLaren & Challis, 2009; White, 2014).

**Fig. 1:**
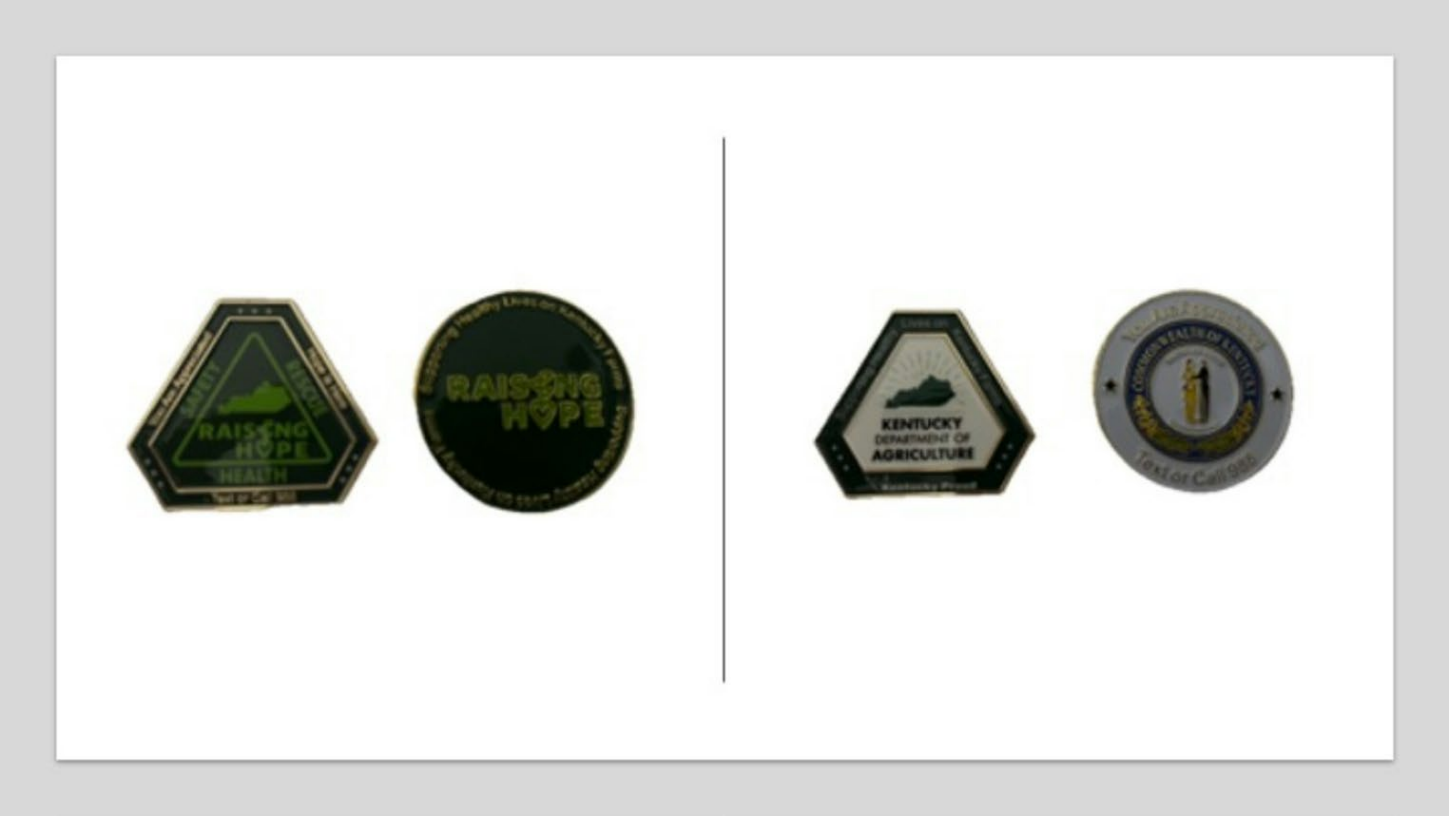
Challenge coin examples

In response to these statistics, a novel suicide prevention intervention was recently developed within a U.S. farming community. This approach adapted the concept of a military-oriented challenge coin, which is a token that the armed forces have used for years among their members in order to develop a sense of belongingness and camaraderie (Lange, 2017). Briefly, in a military context, soldiers or veterans will present a metal token (often imprinted with military imagery such as weapons or unit information) to each other as a signal of a job well done or to show appreciation. Challenge coins have also been used to recognize active duty or veteran members in student populations (Morrison-Beedy & Rossiter, 2018). Leaders of a farming organization in Kentucky, viewing farming as a vital, life-saving service, found that the concept of a challenge coin resonated with many farmers, who have deep connections with the military (Besterman-Dahan et al., 2018, 2023; Census of Agriculture, 2017; Fleming, 2015). Concerned about high suicide rates and a lack of discussion about mental health needs in the agricultural community, these leaders created agriculture-themed challenge coins, adding official seals to the coin and the 988 suicide hotline number (Fig. 1). The coins are presented in an informal conversation between farmers as a caring support tool to encourage farmers to reach out for help with mental health and struggles with suicidal thoughts and behaviors (Ward et al., 2024). Challenge coins were shared preemptively with members of the agricultural community, engaging them in a conversation openly discussing mental health and encouraging them to reach out for help from friends or professionals if they have mental struggles in the future, while sharing appreciation of the recipient due to their vital societal role as a farmer. This community-grown intervention has limited evidence in the scientific literature, so further exploration is needed of the challenge coin as a suicide prevention and caring support tool that fosters belongingness and appreciation among farmers. Currently, this challenge coin initiative is run by a state agricultural department that aims to increase agricultural safety and improve the mental and physical health of farmers. One of the program leaders who initiated the concept of the challenge coin shares the challenge coin with others and trains members of the state’s agricultural communities on the meaning of the coin. In these trainings, the coin’s purpose as a suicide prevention and mental health promotion tool is explained, and language is shared about how to present a challenge coin to others, ensuring that suicide prevention and farmer appreciation are covered in the conversation.

The attributes of the challenge coin indicate that it is an intervention consistent with constructs in the interpersonal-psychological theory of suicide, which considers perceived burdensomeness and thwarted belongingness as suicide predictors (Joiner et al., 2009; Van Orden et al., 2010). Identifying interventions that will increase a sense of belonging and reduce perceived burdensomeness is important to reducing the suicide rate among at-risk individuals. Showing one-on-one appreciation, gratitude, and support for others is protective against suicide (Kleiman et al., 2013; Krysinska, 2018; Maumus, 2022). With this strengths-based approach, the challenge coin takes a comprehensive, community-based public health approach to suicide prevention (Caine et al., 2018), because it is not necessarily given to someone in current distress. Rather, the aim is to instill a sense of appreciation and hope in the recipients, opening the possibility of discussing mental health and stress, and increasing awareness of resources available if distress or crisis situations arise (i.e., 988).

Further, the challenge coin is given by peers in the farming community, ending with encouragement to reach out for mental health assistance in times of anguish, whether that is by calling a friend or seeking professional help, such as a therapist. This encouragement, signified by a sincere handshake between farmers, is not a contract; rather, it is a method to help the individual consider reaching out for mental health assistance during times of distress, and the challenge serves as a tangible reminder for the farmer.

The challenge coin, as adapted for farmers, is a tactic to begin the conversation about mental health, especially in agricultural communities, which historically have stigmatized mental health problems and help-seeking behaviors and have limited access to health care (Becot et al., 2020; Droullard et al., 2017; Hiebert et al., 2016). Direct information from farmers who have recently received the farmer-adapted challenge coin is essential to understand the efficacy of the intervention. Formative research is needed to establish a basis for future evaluations and recommendations for implementation of the intervention. Therefore, this study sought to understand the use of the challenge coin as a suicide prevention intervention among farmers by interviewing recent coin recipients from the Kentucky farming community. The following were the research questions:

- What do the challenge coin recipients remember about the challenge coin presentation?
- How effectively does the challenge coin convey care, support, and an anti-suicide message to the recipient?

## Methods

Participants were purposively sampled based on their characteristics: occupation in farming and recent receipt of a challenge coin from a fellow farmer. The challenge coin was presented individually to all recipients with a short speech about the importance of mental health and appreciation for farmers, encouraging the recipient to reach out for help when they are feeling distressed. Eligibility criteria for this study included being English-speaking, 18 years old or older, working in a farming-related occupation, and receiving a challenge coin within the last six months. The challenge coin program director at the Department of Agriculture and two community leaders were the three main sources of dispensing the challenge coins to recipients. These key informants in sharing challenge coins provided referrals to the first author, who followed up with each potential participant, ensuring eligibility and explaining the study purpose. A telephone or Zoom interview was scheduled with the researcher if they were interested in participating. While none of the participants in this study disclosed suicidal ideation, as that was not a prerequisite for receiving a challenge coin, the researcher was prepared to provide follow-ups to anyone expressing distress during the interviews.

The semi-structured interview guide (Supplementary File 1) provided a basic framework for the interviews, allowing for flexibility in conversation content. Interviews began with questions about the individual’s farming work, establishing curiosity and rapport, with questions about individual stressors and information probes. The challenge coin was introduced, followed by asking individuals open-ended questions about their experiences receiving it, perceptions of the coin, and thoughts about the adapted coin as an intervention intended to protect mental health and prevent suicide in the farming community. While the structure of the interview guide ensured that questions were asked about perceptions of the challenge coin, each participant determined the content of the interview.

Interviews of 20–45 min were conducted from March 2024 to June 2024. Each participant completed informed consent processes before the interview and received a $25 online gift card after the interview in appreciation of their time. The University of Southern California Institutional Review Board approved this study. Interviews were audio-recorded. Demographic data were collected (e.g., age, race, marital status).

After each interview, the recording was uploaded to an encrypted, dual-factor-protected cloud location. Data were deleted from the recorder and transcribed as modified verbatim. The sample size was considered sufficient once the data reached saturation. Demographic data and transcripts were uploaded to the software Dedoose 9.1.012, a web software for handling qualitative and mixed-methods data (Dedoose Version 9.0.17, 2021).

The data were explored by reading full transcripts and debriefing to generate initial ideas. The analysis process was iterative, with three researchers reducing the data into codes and condensing them into groups of information, which were combined into themes to create a codebook (Creswell, 2013). After establishing the themes, the researchers separately coded two transcripts. The researcher and project research specialist compared coding and established intercoder agreement with a minimum of 80% agreement to ensure study rigor (Creswell, 2013). Afterward, the remainder of the interviews were coded separately.

This qualitative study was informed by thematic analysis combined with data coding and analysis, inductively deriving themes and codes from the data. Still, deductive approaches were also used, as the concepts of stressors and coping skills were gathered from the semi-structured interview questions (Braun & Clarke, 2012), along with reactions to the challenge coin concept. Debriefs were written immediately after each interview to engage the researcher in the materials and idea development, and to enable critical reflexivity.

The research team kept reflexivity by maintaining cultural self-awareness and challenging assumptions about farmers. The leading researcher who conducted the interviews has mainly lived in Kentucky and was a cisgender female with no personal farming background, but maintains close relationships with the farming community and has traveled to farm shows, livestock expositions, and farmer health fairs.The coding team members consisted of researchers of Latina/Caucasian and Caucasian descent with backgrounds in public health, psychology, and data analysis, and without experience in farming.

## Results

Fourteen interviews of farmers who received the challenge coin were conducted until the point of saturation, when no new information was shared. Interviewees ranged from age 28 to 68 years (*M* = 49.29, *SD* = 12.86), one participant was female, while the majority were male, and all participants were of Caucasian, non-Hispanic descent. Most of the sample (86%) was married, one was single, and one was divorced.

Although all participants were in farming-related roles, 77% held off-farm jobs, including manufacturing, construction, or administrative roles. Within the previous six months, each participant had received at least one agriculturally-focused challenge coin from someone within the farming community, including peer farmers, high school students involved in Future Farmers of America (FFA) programming in Kentucky, and farming family friends. The challenge coin presentation to each participant included a brief conversation offering appreciation for their occupation and encouragement to reach out for help if they ever faced mental distress, hopelessness, or suicidal thoughts. We identified six major themes (Table 1): three related to the impact of the challenge coin delivery and three from the lasting symbolism of the challenge coin. The themes related to the challenge coin delivery included feelings of hope, gratitude, and honor, the handshake or hug given as the recipient was encouraged to reach out for help in the future, and the relationship between the challenge coin giver and recipient. The themes related to the lasting symbolism of the challenge coin included recognizing the coin as a special item, a prompt to reach out for help, and a reminder of the shared connection with other farmers and community members.

**Table 1:** Themes, subthemes, definitions, and example quotes from challenge coin evaluation

Themes	Subthemes	Definitions	Example quotes
Impact of the challenge coin delivery	Feelings of hope, gratitude, and honor	Participant mentioned positive feelings	“I was very touched” “You can’t help but fill up with a little bit of… But I think it’s hope.” “I was very honored.”
	An impactful delivery about mental health	Participant clearly describes handshake/delivery of coin	“He explained the challenge coin and made you feel good to a good firm handshake with him.” “He said, ‘You carry this with you, and if you ever have a time when you feel like you just, maybe you just need to talk to somebody.’”
	Relationship and role of the challenge coin giver to the farmer	Participant describes how the peer farmer relationship affected their reception of coin	“The only people that can relate to that is somebody who’s done it.” “I don’t think it would mean as much to me if someone random gave it to me; that’s just my thoughts.” “It wouldn’t do anybody any good to receive a challenge coin…somebody that don’t relate to what we do every day”
Ongoing symbolism of challenge coin	A standout or special item	Participant describes how the challenge coin is distinct from other giveaway items	“I’d never throw it away…definitely wouldn’t throw it away. It means something when I see it.” “I got…keep one in my pocket all the time.” “It’s just something to make you stop, think, and appreciate.” “It became a part of me.”
	A prompt to reach out for suicide prevention	Participant describes the challenge coin as a prompt to reach out for mental health help if/when needed	“Anytime that you feel like you’re not, you can look at that coin and flip it over and text or call 9-8-8 to get some reassurance.” “The challenge coin in the end is gonna make you pick up the phone and make that phone call instead of doing something drastic that doesn’t need to happen.” “That coin will save your life if you read it.”
	Finding meaning with other farmers, belongingness, shared struggle, and connectedness	Participant views the challenge coin as a way to ease into conversations about mental health with peers, or the participant is reminded by the coin that they are not alone	“We have a common shared theme in the agriculture industry, so that sometimes can be a conversation starter of going into deeper things.” “There are other people out here who have the same hard job that I do.” “There are people out here that they’re going through the struggles and the stress.” “It lets me know that I’m not alone.”

### Impact of the Challenge Coin Delivery

#### Feelings of Hope, Gratitude, and Honor

Participants were asked about their initial feelings regarding the challenge coin presentation, and the majority disclosed positive emotions such as hope, gratitude, and honor or said they were “emotional” at the time. “I was very touched,” said one farmer (60-year-old), and another shared: “You almost feel like you wanna break down and cry because sometimes you do, but that’s one of the feelings you get pretty emotional” (38-year-old). Hope was a common theme among the recipients of challenge coins, as one participant (68-year-old) shared, “you can’t help but fill up with a little bit of… But I think it’s hope. I think a lot of it’s just a big flash of hope. And the way he [the coin presenter] does it, it’s a big flash of love with it because he means it wholeheartedly. And that means a lot….how can you not be filled up with lots and lots of emotion?” Another discussed their gratitude for the challenge coin and acknowledgment of their shared struggle as a farmer: “I was really thankful for the fact that he gave it to me that I’ve had my struggles just like everybody else” (40-year-old). The challenge coin presentation honored many recipients: “I was very honored. To take that time to, like I said, to be thought of and to give his time and effort to do that and present it to and feel valued enough to be presented that meant a lot” (47-year-old). “It’s kinda hard to explain because you’re full of pride and appreciation” (68-year-old). “I mean, just the way [the coin presenter] took it, and give it to me and explained it to me, that was what… and it’s somebody that you can really believe that’s got just the heart to help someone. And that was what made it mean so much to me” (53-year-old).

#### An Impactful Delivery About Mental Health

One recollection of the challenge coin presentation was: “He said, ‘I got something I wanna give you.’ So he pulled a coin out, and he said, ‘Now this is a challenge coin.’ He went over it and, talking about, ‘Hey, this says that you’re appreciated.’ And showed me the nine-eight number, and he said, ‘You carry this with you, and if you ever have a time when you feel like you just, maybe you just need to talk to somebody.’ And offered it to me. And he said, ‘I offer to you with a handshake. If you want a hug, brother, I’ll give you one, but if you get the hug, you gotta promise me that you’ll always call this number before you do anything’” (53-year-old). Another description of the challenge coin presentation (47-year-old) elaborated on feeling cared for and valued: “He explained the challenge coin and made you feel good to a good firm handshake with him. To pull you in, tell you he cares, and take time to truly check on you and find out what’s going on in your life to… and present you with a coin to help remember that somebody cares and that you’re valued. And I guess respected that way enough in the profession of agriculture and farming here to…To bring that recognition about… meant a lot, and means a lot today, thinking back on it.”

#### The Relationship and Role of the Challenge Coin Giver to the Farmer

It mattered to these farmers that their challenge coins were from someone with a relationship with the farming community; as one farmer put it: “The only people that can relate to that is somebody who’s done it” (38-year-old). Most challenge coin recipients knew the coin givers for many years, although some were more recent acquaintances. “I don’t think it would mean as much to me if someone random gave it to me; that’s just my thoughts” (64-year-old). Another commented, “you don’t have to be best friends with ‘em, but I think there needs to be some relationship prior there. I think if some random person walks through and hands you a coin, I just don’t think it’s gonna mean something like somebody that’s actually gotten to know you a little bit” (35-year-old). “I feel more like he understands what I would be going through,” said another interviewee (35-year-old). “It wouldn’t do anybody any good to receive a challenge coin….somebody that don’t relate to what we do every day and what we gotta do…. to know that somebody give you a challenge coin come from that type of lifestyle and things, that was pretty neat,” continued one interviewee (38-year-old). “There ain’t no better, better than [COIN-GIVER] for this job. ‘cause he’s been a farmer his whole life too” (35-year-old). “I think it’s… that bond there of being in another person’s shoes like that and understand where they’re coming from. It meant more that way” (47-year-old). “To get it from some stranger that’s… I know the meaning of it, but that’s not the same. That’s not the same….knowing that person and knowing how that person thinks and believes it’s what really puts the meaning in it…. it means more when it comes from somebody you know that basically has a passion for the same industry you have….I think the value of a farmer challenge coin is significant when you’re talking to a farmer. And that’s huge” (68-year-old). While several of the recipients optimistically suggested that the challenge coin could be given by anyone, most believed that the challenge coin was best received from a fellow farmer or someone with a firsthand understanding of the day-to-day stressors.

The power dynamic of the challenge coin giver was also important, as it was more impactful coming from a peer rather than a student or someone much younger. While a challenge coin given by an FFA student or young adult to an older adult was well-received, the recipients’ attitudes were more dismissive, as the challenge coin intent was more powerful coming from a peer who had life experience as a farmer. For example, one participant shared: “I knew [their parents] from [redacted]. I said, well, ‘why don’t you give that to somebody else that would have some problems?… I said, well, ‘if this helps you, I will take that.’”.

### After the Presentation: Ongoing Symbolism of the Challenge Coin

#### The Challenge Coin Was a Special, Standout Item to the Recipients: “No. I’d Never Throw It Away”(60-year-old)

Interviews were completed months after the coin had been received by the participants. Importantly, every participant knew where their challenge coin was, as most had it in their pocket, desk, dresser, or lunchbox. It was considered a standout item, something meaningful and special that they would not throw away, differentiating it from other trinkets: “definitely wouldn’t throw it away. No. It means something when I see it.” (47-year-old). “I got keep one in my pocket all the time. And you can reach in to get some coins, and you always feel the big coin. And it makes you smile. It makes you think a second. And that’s what it’s all about. It’s just something to make you stop, think, and appreciate,” said one farmer (68-year-old). One farmer relayed that “it became a part of me” (45-year-old). It was considered a standout item, given its size and weight: “I think when you give that, it’s such a significant thing, they’re not gonna throw it in the dash of the truck or throw it away or doing that. It’s something that looks important” (52-year-old).

#### The Challenge Coin Provides a Prompt to Reach Out for Suicide Prevention

Many farmers described being reminded of hope for future generations when they looked at the coin, noting the message of appreciation and the reminder to reach out for help. “And with the coin itself, anytime that you feel like you’re not, you can look at that coin and flip it over and text or call 9-8-8 to get some reassurance,” shared one farmer (64-year-old). The farmers appeared to understand the purpose of the 988 Suicide Hotline and the suicide prevention goal of the challenge coin: “the text or call 9, 8, 8 shows that there is a basically a hotline that we can reach out to…. in case times get rough and I, or someone, with a coin thinks they don’t have a place to turn,” explained a recipient (64-year-old). Another recipient (35-year-old) shared: “It gives you another lifeline to just call if you ever do… hopefully that doesn’t ever cross my mind, but I think if it does, it tells you to say somebody truly appreciates you and there’s no better feeling than knowing that you’ve got somebody…. the challenge coin in the end is gonna make you pick up the phone and make that phone call instead of doing something drastic that doesn’t need to happen.” “It is just a reminder that, ‘hey, stop thinking and call and talk to somebody.’ There is people that’ll talk,” another farmer (35-year-old) said, referring to the ability to reach out to someone at any time. “Having the coin given to me definitely brought into perspective, ‘Hey, you need to go get help before’…. the thoughts are always, were there constantly,” another farmer (40-year-old) shared. “That coin will save your life if you read it,” another farmer (62-year-old) shared.

#### The Challenge Coin Helps Participants Find Meaning With Other Farmers, Belongingness, Shared Struggle, and Connectedness

The farmers interviewed considered the challenge coin to be a way to ease into conversations about mental health among their peers: “we have a common shared theme in the agriculture industry, so that sometimes can be a conversation starter of going into deeper things” (28-year-old). “Having something there to me breaks the ice,” explained a farmer (38-year-old). One farmer described the challenge coins as “the conversation piece that gets it started “ (68-year-old), referring to mental health conversations.

The challenge coin recipients stated that the coin served as a reminder of “having that connection with people that touch, that handshake especially, for a hug, whichever the case may be just truly telling somebody that, ‘yes, you’re not alone and we’re here for you. It’s okay” (40-year-old). “I think for a lot of us, it hits home better. It’s closer to home. Because we’re all dealing with a lot of the same stress” (28-year-old). “I know that there are people out here that care enough about farmers and their wellbeing and what they’re doing as a job” (35-year-old). “for me, it’s, it knows that you’re not in this alone. There are other people out here that are farming. There are other people out here who have the same hard job that I do. There are people out here that they’re going through the struggles and the stress and everything. And if you need, you get in a place where you just, I mean, everybody gets in a dark part of their mind every now and then when things just aren’t going right.” (35-year-old). “It lets me know that I’m not alone,” shared another farmer (60-year-old). “When they find out that the other people have the problem and can talk to people, I think that’s a big relief to them….we’re all in this, we’re all in this together….These challenge coins kind of just reiterate the fact that you’re not in this boat alone. Everybody has problems. And it’s just good to admit, or not to admit, but just to open up and tell you problems. And other people have the same situation” (63-year-old).

## Discussion

This study focused on the feelings, thoughts, and interpretations of farmers who received a challenge coin tailored to the farming profession and intended as a suicide prevention intervention. Receiving the challenge coin was a positive experience for all the participants, but the response was positively amplified given a shared farming identity with the coin giver. Other studies corroborate this finding, as they identify that farmers trust their peers over experts (Joffre et al., 2020; Rust et al., 2022). Further, the results described in this study suggest recommendations for optimized presentation of the challenge coin in the agricultural community. A relationship with the giver of the coin is necessary, and the giver needs to know the challenges faced by the farmer community. Studies have emphasized the strong role that peers in subgroups can take in suicide prevention interventions (Bowersox et al., 2021; Van Orden et al., 2013), and those dynamics emerged prominently in this sample of farmers. Indeed, peer support is an effective method for farmers to engage with their counterparts and increase their mental well-being (Kaur et al., 2022; Nye et al., 2023; Saju et al., 2024).

While 988 is imprinted on the coin, it is not specifically described as a suicide hotline on the coin, necessitating the context in the presentation of the challenge coin (i.e., the presenter explains 988 to the recipient). Thus, when groups learn about the concept of challenge coins and their attributes, they need to ensure that mental health intervention intent is mentioned and that 988 is explained as the National Crisis Lifeline. Compared to the general population, farmers have a higher rate of suicide but lower rates of diagnosed depressive disorders (Daghagh Yazd et al., 2019), suggesting that healthcare-based suicide prevention may not have as much reach for farmers. Further, they are geographically isolated with access to lethal means (Miller & Rudolphi, 2022), and express less suicidal ideation (Reed & Claunch, 2020), complicating timely outreach to farmers who are potentially in acute suicidal crises. Therefore, having an on-hand cue, such as the challenge coin, for the farmer in crisis may encourage them to reach out to the National Crisis Lifeline or remind them of a trusted connection and the handshake they made upon receipt of the coin.

Most importantly, among the participants, the challenge coin nearly uniformly symbolized appreciation toward agriculture and a reminder to reach out for mental health assistance during challenging times. The challenge coin presentations effectively expressed gratitude toward and increased belongingness and social connectedness in farmers. Interventions that involve increasing belongingness and reducing individuals’ perception that they are a burden on others or that invoke gratitude are strategies to lower suicidal ideation (Chu et al., 2017; Gill et al., 2023; Kaniuka et al., 2021; Kleiman et al., 2013). All of the participants in this study recognized the value of the challenge coin for suicide prevention. While this is the first exploration of a challenge coin adapted as a suicide prevention intervention in farmers, this study helps establish that farmers found meaningful interaction in the intervention. Additional research in more populations is needed regarding the impact of the coin and ways in which facilitator effects (i.e., the person presented the coin to the recipient) can be documented to enhance training in and scaling up this kind of community-based intervention.

In a public health approach to suicide prevention, community-based interventions continue to be integral strategies (Caine et al., 2018), but are less frequently studied and less resourced than clinically-focused interventions. As an example of this imbalance, the Action Alliance alongside the National Institute of Mental Health, conducted a portfolio analysis of research funding for suicide prevention, finding that only 13 of 383 reviewed suicide prevention studies focused on prevention or intervention outside of clinical settings, a stark contrast to 43 of 383 studies that focused on biomedical and psychotherapeutic interventions (The Action Alliance, 2015). Community-initiated interventions, such as the challenge coin in the present analysis, arise outside of an academic- or medical-led model and often begin at places that seem trivial. For example, community gardens are evidence-based interventions that can increase social connectedness (Tracey et al., 2023). Similarly, a coin may seem like a trivial object around which to form an intervention. However, the present findings suggest that the coins made a potentially lasting impression on the recipient, as most participants knew where the coin was stored weeks or months after having received it. Consequently, explorations such as this study of a community-initiated effort reify the ways in which academic and biomedical researchers can learn from community members in terms of tailoring outreach.

Because of the relatively small, tight-knit communities of farmers in which coins were dispensed and, in some cases knowing the coin presenter, some participants’ reactions may have had facilitator effects and potential social desirability bias. For instance, one participant described the coin giver as “somebody that you can really believe that’s got just the heart to help someone” (53-year-old). This statement suggested the influence of the intent behind the conversation and that the recipient may have been equally impressed by the facilitator as the challenge coin. Another farmer stated about the coin-giver that “there ain’t no better, better than [COIN-GIVER] for this job. ‘cause he’s been a farmer his whole life too” (35-year-old), suggesting the social desirability and influence of the facilitator. This facilitator effect may have influenced participants to provide more favorable answers to questions, presenting a more positive narrative of their viewpoints than they genuinely believe (Latkin et al., 2017). More research on the challenge coin validity is needed, examining the effects of diverse challenge coin-givers on recipients’ perceptions and whether other promotional items would hold symbolism similar to the challenge coin.

There were several other limitations to this study. Because qualitative work is not intended to be generalizable, the results may not extend from the participants in this specific US State. The purposive sampling strategy resulted in a lack of racial and gender diversity in the sample. However, it should be noted that the farming producer population of Kentucky is largely comprised of individuals who are white (99% of producers) and male (65%) (U.S. Department of Agriculture, 2022). The scope of the interviews was limited to the experience of receiving a coin, and other information potentially pertinent to suicide prevention topics (e.g., mental health care utilization, surviving a loss to suicide) was not explored in the interviews given time and resource constraints. Further, although one researcher has been embedded in the farming community and maintains relationships with multiple farming community members, as this study did not receive data checking, trustworthiness may be limited, given that none of the authors is also a farmer.

## Conclusions

Community-driven interventions are increasingly important avenues for a public health approach to suicide prevention (Caine et al., 2018), and this study explored a locally-initiated, tailored intervention implemented with farmers in Kentucky. The intervention of an agriculture-adapted challenge coin for suicide prevention was well-received by the farmers sampled in this study, who found it a powerful way to start much-needed conversations about mental health in traditionally stoic communities. More research is needed on the impact of the challenge coin adapted for agricultural suicide prevention, and the presentation behind the challenge coin requires planning and training to ensure that the messaging about suicide prevention is preserved. However, this is a promising intervention to open communication channels about mental health in this community that is at-risk for suicide, with an item that reminds farmers that they are not alone, and that they have peers and resources to turn to during times of despair.

## Supplementary Information

Below is the link to the electronic supplementary material.

Supplementary file 1 (DOCX 24.0KB)
