# Supplementary material for: “Stop, Think, and Appreciate”: A Qualitative Exploration of a Challenge Coin Suicide Prevention Intervention among Farmers
Source: Community Ment Health J. 2025 Aug 20;62(1):145–54. doi: 10.1007/s10597-025-01509-1 (PMC12789155; doi:10.1007/s10597-025-01509-1)
Supplement: Supplementary file 1 — (DOCX 24.0KB) [file 10597_2025_1509_MOESM1_ESM.docx]

**Challenge Coin Study**

**Qualitative Interview**

**Interview domains and examples of broad interview questions**

| **Occupation and challenge coin receipt** | |
| --- | --- |
| *Question* | *Example Participant Questions [probe/follow-up]* |
| Profession | What is your job title/duties?  What are some stressors in your role?  How do you deal with these? How do your peers? |
| Challenge coin memory | Tell us about receiving the challenge coin.  How did you feel when you received it?  Where is it now? Where do you usually keep it? |
| Participant’s experience with challenge coins | Had you ever heard of challenge coins before receiving this one? [e.g. police/military/first responder/government leadership]  If yes: what makes this challenge coin different? |
| **Challenge coin receipt experience** | |
| Participant Relationship | How would you describe your relationship/role with the giver of the challenge coin at the time of disclosure? |
| Participant Observation | What do you remember about the conversation you had about the challenge coin?  What stands out to you? |
| Participant’s Reaction | When you were given the challenge coin, what was your first thought that you remember? Emotion? |
| Participant’s Response | What did you first say or do after receiving the challenge coin?  Tell us how you felt. [Was it special?]  How does the challenge coin stand out to you as a special possession or not? [Why wouldn’t you throw it away like another giveaway? Or why did you throw it away?] |
| Participant Help-seeking | Where do you think you’d seek help for mental health if you needed it? [call a friend, call 988, seek a therapist etc.]  To whom have you reached out to for mental health support since receiving the challenge coin, if anyone?  If you were to have mental health trouble, is [the giver of the coin] someone you would call or text first for support? Who would you reach out to first?  How does the challenge coin affect your perception of mental health, if at all? |
| Social Media/Internet Searches | What social media/Internet search engines have you used, if any, to learn more about mental health since receiving a challenge coin? |
| **Factors related to suicidal ideation** |  |
| Participant’s knowledge of suicidal risk | What behaviors (verbal or non-verbal) are associated with suicidal thoughts? |
| Connectedness/belongingness | What makes you feel more connected to your community?  What makes you and other people in your industry feel like they belong to a community?  How does the challenge coin support people in your industry?  How does the challenge coin make you feel more connected to others, if at all? |
| Appreciation/burdensomeness | Please share what makes you feel appreciated in your role.  What makes you feel burdened?  What about the challenge coin and/or its presentation, if anything, makes you feel more appreciated or burdened? |
| Recommendations Regarding Suicide Prevention Resources | If you met a fellow farmer who was having thoughts about suicide, where would you advise them to find information or help? Where would you advise them to be cautious about going [such as organizations, social media sites]? |
| **Previous training in mental health** | |
| Prior training for suicide prevention | Have you ever received training about suicide prevention? If so, can you briefly describe that training – how was it delivered, where was it delivered. How often have you received training? (for example, yearly, only once) |
| Other questions | Is there anything else you would like to mention in regards to the challenge coin and farmer mental health? |

| **Demographics** | |
| --- | --- |
| Age | What is your current age? |
| Gender Identity | What is your gender identity?  *Probe:*  *If respondent needs clarification, read: a. cisgender man; b. cisgender woman; c. transgender man; d. transgender women; e. nonbinary/gender nonconforming*  *If participant asks clarification of cisgender and transgender, explain that cisgender means that the sex assigned to you at birth is the gender you currently identify with and transgender means that you identify as a gender identity different than the sex assigned to you at birth.* |
| Race | How would you describe your race? |
| Ethnicity | How would you describe your ethnicity? |
| Relationship status | What is your current relationship status?  *Probe:*  *If respondent needs clarification, read: a. partnered or married; b. separated or divorced; c. widowed; or d. single* |
